# Randomized, multicenter phase 3 study evaluating radiotherapy versus concurrent chemoradiotherapy in nasopharyngeal carcinoma patients achieving CR/PR after induction chemotherapy

**DOI:** 10.1186/s12916-026-05064-8

**Published:** 2026-07-11

**Authors:** Ting Jin, Feng Jiang, Qi-feng Jin, Yong-Hong Hua, Yong-shi Jia, De-xi Du, Shu-bo Ding, Hua-fang Su, Miao-zhen Lu, Hui-juan He, Xiao-zhong Chen

**Affiliations:** 1 Department of Radiation Oncology, Zhejiang Cancer Hospital, Hangzhou Institute of Medicine (HIM), Chinese Academy of Sciences, Hangzhou, Zhejiang 310022 China; 2 Key Laboratory of Head & Neck Cancer Translational Research of Zhejiang Province, Hangzhou, 310022 China; 3 Department of Radiation Oncology, Zhejiang Provincial People’s Hospital, Hangzhou, Zhejiang 310014 People’s Republic of China; 4 Department of Radiation Oncology, The Central Hospital of Lishui City, Lishui, Zhejiang 323000 People’s Republic of China; 5 Department of Radiation Oncology, Jinhua Central Hospital, Jinhua, Zhejiang 321000 People’s Republic of China; 6 Department of Chemoradiation Oncology, The First Affiliated Hospital of Wenzhou Medical University, Wenzhou, Zhejiang 325000 People’s Republic of China; 7 Department of Chemoradiation Oncology, Ningbo Medical Center Lihuili Hospital, Ningbo, Zhejiang 315000 People’s Republic of China; 8 Department of Radiation Oncology, People’s Hospital of Quzhou, Quzhou, Zhejiang 324000 People’s Republic of China

**Keywords:** Nasopharyngeal carcinoma, Induction chemotherapy, Radiotherapy, Concurrent chemoradiotherapy, Tumor response

## Abstract

**Background:**

The present study sought to compare the clinical efficacy and safety profiles of radiotherapy (RT) and concurrent chemoradiotherapy (CCRT) in nasopharyngeal carcinoma (NPC) patients who attained complete or partial response (CR/PR) following induction chemotherapy (IC).

**Methods:**

This multicenter, randomized, open-label, phase 3 noninferiority trial was conducted at 7 Chinese hospitals. Patients with newly diagnosed stage III-IVb NPC who achieved CR/PR after IC were randomly allocated (1:1) to either the RT (IC + RT) group or the CCRT (IC + CCRT) group. The chemotherapy protocol was identical across the two treatment groups. The primary endpoint was progression-free survival (PFS). Secondary endpoints comprised overall survival (OS) and the incidence of adverse events. Statistical analyses were performed using the Kaplan-Meier method and log-rank test.

**Results:**

From December 2016 to May 2023, 220 eligible patients with histologically confirmed nasopharyngeal carcinoma were enrolled. The trial was terminated early due to slow accrual. Of these patients, 109 were assigned to the IC + RT group and 111 to the IC + CCRT group. The median follow-up was 57 months (range, 8-107 months). The IC + RT and IC + CCRT groups showed comparable 5-year progression-free survival rates (87.0% vs. 80.7%, *P* = 0.21). No significant differences were observed between the two treatment arms with respect to local relapse-free survival, regional relapse-free survival, locoregional relapse-free survival, distant metastasis-free survival, or overall survival (all *P* > 0.05). The IC + RT group had significantly lower incidences of grade 3–4 hematologic and gastrointestinal toxicities than the IC + CCRT group. Additionally, grade 1–2 deafness/otitis and liver dysfunction were also less frequent in the IC + RT group.

**Conclusions:**

In NPC patients achieving CR/PR after induction chemotherapy, IC + RT demonstrated a favorable safety profile compared with IC+CCRT. Although the observed survival outcomes were numerically comparable, the premature termination of the trial and insufficient statistical power preclude a definitive noninferiority claim. These hypothesis-generating findings warrant validation in future adequately powered trials.

**Trial registration:**

ClinicalTrials.gov, NCT03015727.

**Supplementary Information:**

The online version contains supplementary material available at https://doi.org/10.1186/s12916-026-05064-8.

## Background

In newly diagnosed nasopharyngeal carcinoma (NPC), locally advanced (LA) cases account for two-thirds. Compared to early and intermediate stages, patients with locally advanced NPC have a poor prognosis and require combined chemoradiotherapy [1].

The National Comprehensive Cancer Network (NCCN) recommends induction chemotherapy followed by concurrent chemoradiotherapy for patients with T3N0M0 nasopharyngeal carcinoma accompanied by high-risk characteristics, including bulky primary tumor volume and increased serum EBV DNA copy number. In addition, for patients with T3N1-3M0, T4N0-3M0, or T0 (EBV-positive)-2N2-3M0 disease, IC + CCRT is considered the preferred treatment paradigm in the NCCN guidelines [2]. Unfortunately, IC + CCRT is associated with high acute adverse events and low completion rate of concomitant chemotherapy (CC). More than 70% of patients experience grade 3 or higher acute adverse events, and only about half of the patients complete the originally planned 3 cycles of CC [3–5]. CC is one of the main causes of these side effects [6]. Therefore, the key question is whether CC is necessary for all LA NPC patients. Previous studies have demonstrated that patients who achieved complete response (CR)/partial response (PR) after IC had a better prognosis than those with stable disease (SD)/progressive disease (PD) [7–10]. Consequently, patients with CR/PR after IC may be optimal candidates for omitting CC.

This multicenter, randomized, open-label, phase III trial was initiated to compare the efficacy and toxicity of radiotherapy (RT) versus CCRT in patients with locoregionally advanced nasopharyngeal carcinoma who achieved CR/PR following induction chemotherapy.

## Methods

### Study design and participants

This randomized, open-label, phase 3, noninferiority trial was conducted at 7 Chinese hospitals between December 1, 2016, and May 31, 2023. Eligibility criteria included newly diagnosed, histologically confirmed non-keratinizing carcinoma; T3-4 and/or N2-3 disease based on the 7th AJCC staging system; no evidence of distant metastasis; Karnofsky performance status ≥ 70; adequate liver, renal, and hematologic function, as indicated by alanine aminotransferase (ALT) and aspartate aminotransferase (AST) < 1.5 × the upper limit of normal, creatinine clearance > 60 mL/min, white blood cell count > 4 × 10⁹/L, hemoglobin > 80 g/L, and platelet count > 100 × 10⁹/L; controlled blood glucose in patients with diabetes; written informed consent; and a favorable response to induction chemotherapy, defined as CR or PR. Tumor response to CR or PR was assessed according to the Response Evaluation Criteria in Solid Tumors (RECIST) version 1.1. CR was defined as the disappearance of all target lesions and reduction of lymph node short-axis diameter to < 10 mm. PR was defined as a ≥ 30% decrease in the sum of diameters of target lesions compared with baseline.

Patients were excluded if they had WHO type I squamous cell carcinoma or adenocarcinoma, were younger than 18 years or older than 65 years, had a history of renal disease, had a previous malignancy other than adequately treated cervical carcinoma in situ or basal/squamous cell skin cancer, had received prior chemotherapy or radiotherapy unrelated to non-melanoma skin cancer outside the intended irradiation field, were pregnant or breastfeeding, or had peripheral neuropathy or significant psychological disorders. The trial protocol was approved by the Ethics Committee of Zhejiang Cancer Hospital (approval number: IRB-2016-057). All procedures were conducted in accordance with the 1964 Helsinki declaration and its later amendments.

### Randomization and masking

Randomization was performed at the Zhejiang Cancer Hospital using computer-generated random number codes. Patient assignment was performed using 1:1 randomization. This was an open-label trial; therefore, no masking was implemented.

### Procedures

**Fig. 1:**
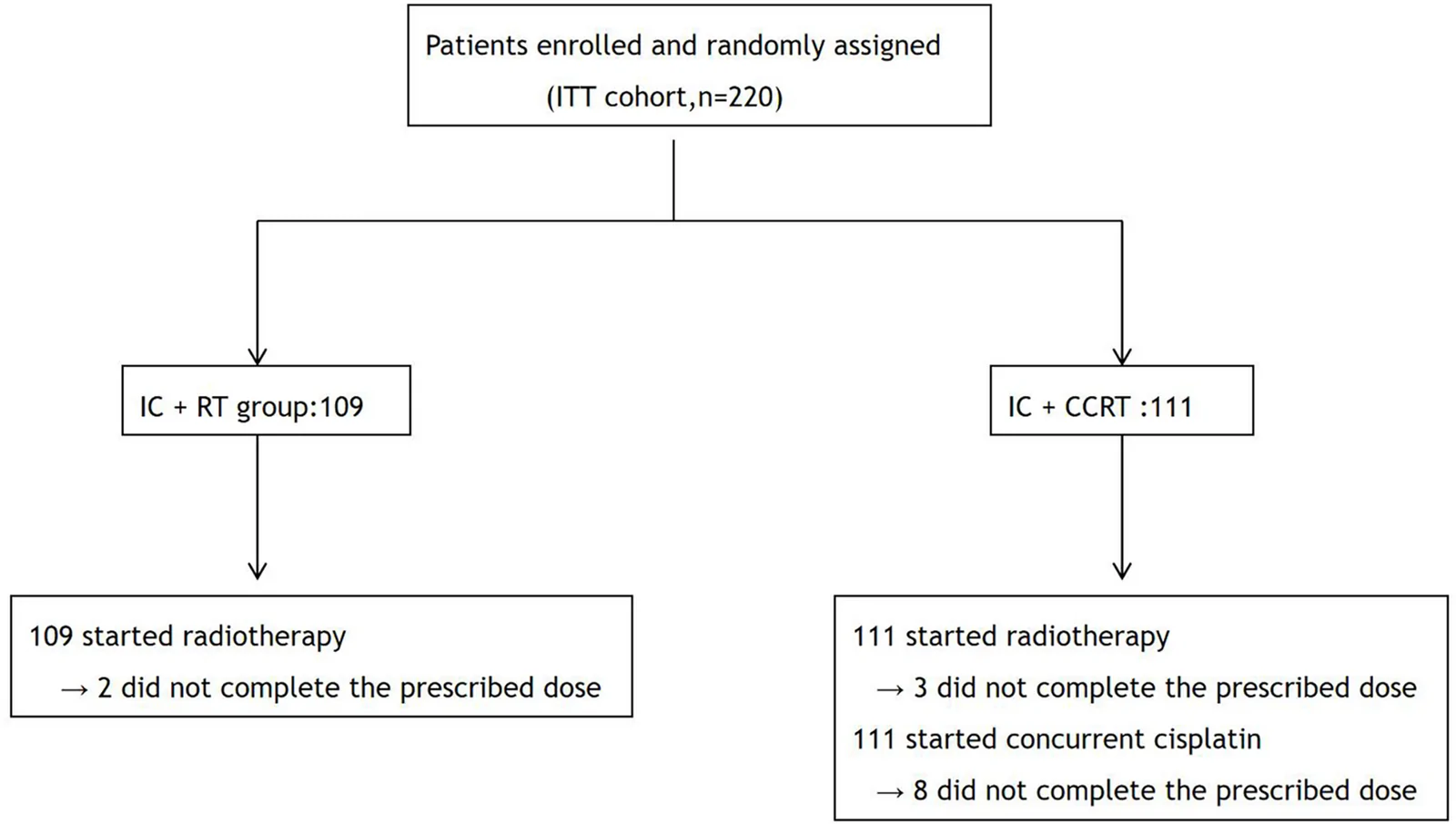
Study profile. ITT, intention-to-treat; IC + RT, induction chemotherapy plus radiotherapy (RT); IC + CCRT, induction chemotherapy plus concurrent chemoradiotherapy

Patients who attained CR or PR after three cycles of induction chemotherapy were randomly assigned in a 1:1 ratio to the RT group or the CCRT group (Fig. 1). Intensity-modulated radiotherapy (IMRT) was delivered during the radiotherapy phase, with a total prescribed dose of 69.96 Gy in 2.12 Gy per fraction to the planning target volume of the gross disease. Patients in the CCRT arm received two additional cycles of concurrent cisplatin on days 64 and 85 at a dose of 100 mg/m².

### Outcomes

The primary endpoint was progression-free survival (PFS), defined as the time from randomization to the first occurrence of disease progression, including locoregional relapse, distant metastasis, or death from any cause. Secondary endpoints included overall survival (OS), local relapse-free survival (LRFS), regional relapse-free survival (RRFS), locoregional relapse-free survival (LRRFS), distant metastasis-free survival (DMFS), and toxicity. OS was defined as the time from randomization to death from any cause. Adverse events, including but not limited to mucositis and hepatic or renal impairment, were assessed and documented throughout induction chemotherapy, concurrent chemoradiotherapy, and follow-up according to the National Cancer Institute Common Terminology Criteria for Adverse Events (NCI-CTCAE) version 3.0.

### Sample size calculation

The IC + RT group was designated as the experimental group, and the IC + CCRT group served as the control group. This trial aimed to assess whether the 3-year PFS in the IC + RT group was noninferior to that in the IC + CCRT group. The 3-year PFS was estimated for the IC + CCRT group to be 85%, based on previously published data demonstrating that patients with locoregionally advanced nasopharyngeal carcinoma who achieved a favorable tumor response (CR/PR) after neoadjuvant chemotherapy had 3-year PFS rates of approximately 85% [7]. The noninferiority margin was established at 10%. To demonstrate the noninferiority of IC + RT with a 5% one-sided significance and 80% statistical power, 400 patients (200 per cohort) were needed. Considering a 10% loss to follow-up, inclusion of 440 patients was required (220 per cohort).

### Statistical analysis

Categorical variables, including adverse events, were compared using the chi-square test, continuity-corrected chi-square test, or Fisher’s exact test, as appropriate. Survival outcomes were estimated by the Kaplan-Meier method and compared between groups using the log-rank test. Due to early trial termination and failure to achieve the target sample size (220/440, 50%), the study was underpowered for the planned noninferiority test. Consequently, the primary analysis was redefined as exploratory. Statistical analyses were performed with SPSS version 21.0 (IBM Corp., Armonk, NY, USA). All reported P values were two-sided, and statistical significance was defined as *P* < 0.05.

## Results

### Patients and treatment

From December 1, 2016, to May 31, 2023, we enrolled 220 eligible patients who were randomly assigned to the IC + RT (*n* = 109) or the IC + CCRT group (*n* = 111). Due to slow enrollment, this study was terminated before reaching the target sample size. The two groups showed balanced baseline characteristics except for sex and clinical stage (Table 1). Compared with the IC + CCRT group, there were more female (36.7% vs. 20.7%) and stage III (72.5% vs. 55.9%) patients in the IC + RT group. To explore the potential impact of these imbalances on survival outcomes, we performed a post hoc propensity score matched analysis (Additional file 1: Table S1). After matching, the survival results remained consistent with the primary analysis, showing no significant differences between groups (Additional file 2: Fig. S1). Radiotherapy was successfully completed during the treatment period in 107 patients (98.2%) in the IC + RT arm and 108 patients (97.3%) in the IC + CCRT arm. Among patients who did not complete the full prescribed radiotherapy dose, one patient in the IC + RT group received 63.6 Gy and one received 67.84 Gy. In the IC+CCRT group, one patient received 61.48 Gy and two patients received 63.6 Gy. During radiation, two cycles of concomitant chemotherapy were completed by 103 patients (92.8%) in the IC + CCRT group. Among the eight patients in the IC+CCRT group who did not complete the planned two cycles of concurrent chemotherapy, five discontinued due to adverse events related to chemoradiotherapy (primarily grade 3–4 hematologic or gastrointestinal toxicities), and three refused further treatment.

**Table 1:** Baseline characteristics of the 220 patients with locoregionally-advanced nasopharyngeal cancer in each treatment arm

Variable	IC + RT (*n* = 109)	IC + CCRT (*n* = 111)	*P*-value*
Sex			**0.009**
Male	69 (63.3)	88 (79.3)	
Female	40 (36.7)	23 (20.7)	
Age, years			
Median	57	52	
Range	20–65	23–65	
EBV-DNA			0.555
0-500	70 (64.2)	67 (60.4)	
>500	39 (35.8)	44 (39.6)	
T category			0.362
T 1–2	14 (12.8)	10 (9.0)	
T 3–4	95 (87.2)	101 (91.0)	
N category			0.418
N 0–1	51 (46.8)	58 (52.3)	
N 2–3	58 (53.2)	53 (47.7)	
Stage			**0.010**
III	79 (72.5)	62 (55.9)	
IVA-B	30 (27.5)	49 (44.1)	
Response to Neoadjuvant Chemotherapy			0.778
CR	10 (9.2)	9 (8.1)	
PR	99 (90.8)	102 (91.9)	

*Calculated using the χ² test. Values are shown as *n* (%) . Statistically significant P values (<0.05) are shown in bold

### Efficacy

**Fig. 2:**
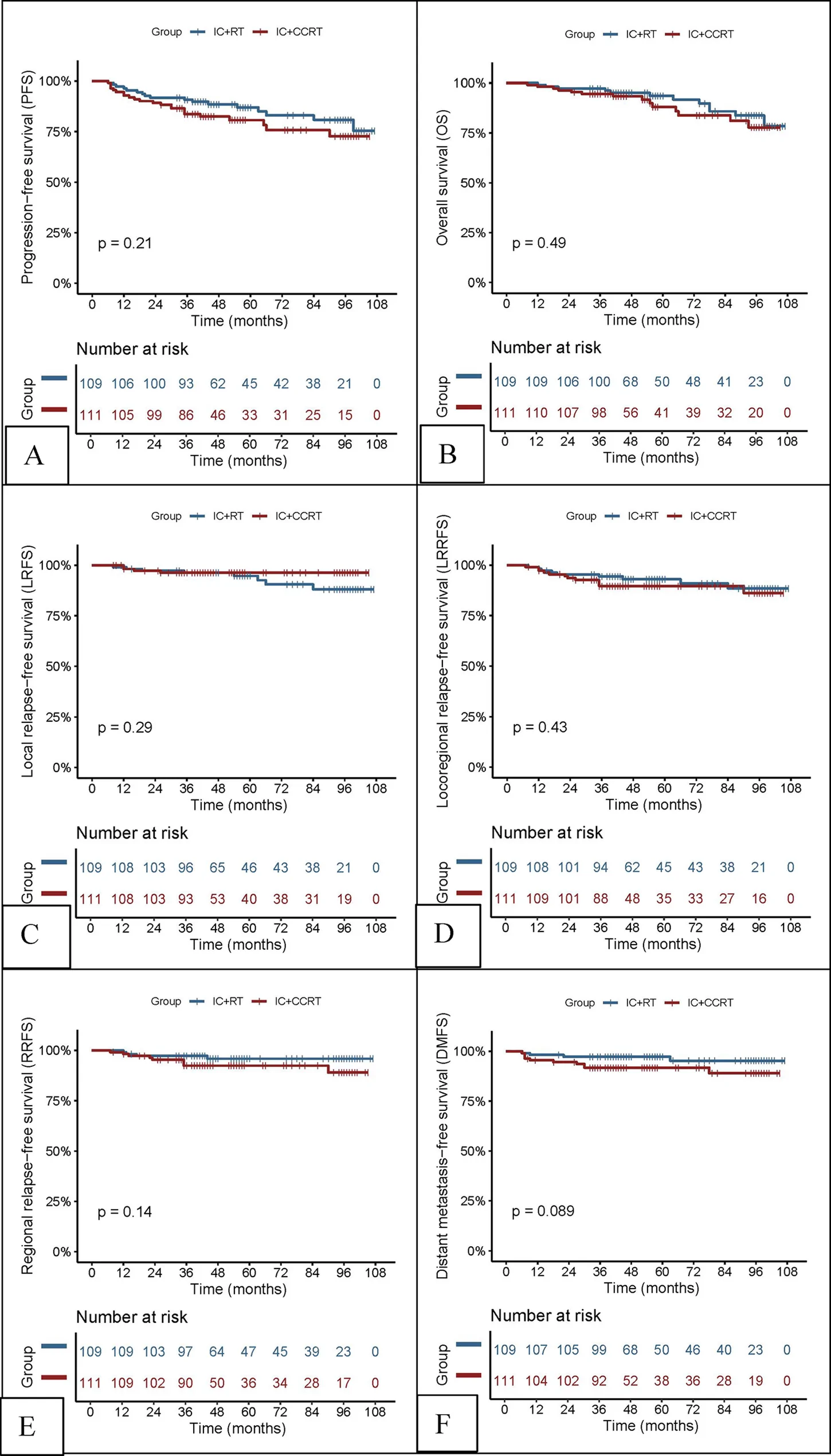
Kaplan-Meier estimates of (**A**) Progression-free survival for patients receiving IC + RT and IC + CCRT (*p* = 0.21). The IC + RT group curve is consistently above the IC+CCRT curve; however, due to the insufficient sample size (50% of planned enrollment), a formal noninferiority test was not conducted, and definitive conclusions cannot be drawn. **B** Overall survival for patients receiving IC + RT and IC + CCRT (*p* = 0.49). **C** Local relapse-free survival for patients receiving IC + RT and IC + CCRT (*p* = 0.29). **D** Locoregional relapse-free survival for patients receiving IC + RT and IC + CCRT (*p* = 0.43). **E** Regional relapse-free survival for patients receiving IC + RT and IC + CCRT (*p* = 0.14). **F** Distant metastasis-free survival for patients receiving IC + RT and IC + CCRT (*p* = 0.089)

At the final follow-up on December 31, 2025, the median duration of follow-up for the overall cohort was 57 months (range, 8-107 months). The 5-year PFS rate was 87.0% in the IC + RT group and 80.7% in the IC + CCRT group, with no statistically significant difference between the two groups (*P* = 0.21; Fig. 2A). The between-group difference in 5-year PFS (IC + RT minus IC+CCRT) was + 6.3% points, with a 95% confidence interval (CI) ranging from − 3.4% to + 16.0%. Although the point estimate fell within the prespecified noninferiority margin of 10%, the wide confidence interval—attributable to the limited sample size (50% of planned enrollment)—crossed the noninferiority boundary, precluding a definitive claim of noninferiority. With respect to the secondary efficacy endpoints, the 5-year OS, LRFS, RRFS, LRRFS, and DMFS rates in the IC + RT group were 93.7%, 94.7%, 95.9%, 93.0%, and 97.2%, respectively. The corresponding rates in the IC + CCRT group were 88.1%, 96.3%, 92.7%, 89.7%, and 91.8%, respectively. No significant differences were identified between the two treatment groups for any of the secondary endpoints (Fig. 2B and F; all *P* > 0.05).

### Safety

The most frequently observed grade 3–4 hematologic adverse events were leukopenia (8 of 109 [7.3%] in the IC + RT group vs. 28 of 111 [25.2%] in the IC + CCRT group), neutropenia (7 [6.4%] vs. 26 [23.4%]), thrombocytopenia (4 [3.7%] vs. 13 [11.7%]), and anemia (4 [3.7%] vs. 12 [10.8%]) (Table 2). Among non-hematologic grade 3–4 toxicities, the most common events were stomatitis (16 of 109 [14.7%] in the IC + RT group vs. 33 of 111 [29.7%] in the IC + CCRT group), nausea (2 [1.8%] vs. 24 [21.6%]), and vomiting (1 [1.0%] vs. 22 [19.8%]) (Table 2). All these differences were statistically significant (all *P* < 0.05). Grade 3–4 late adverse events occurred in 16 (14.7%) and 21 (18.9%) patients in the IC + RT and IC + CCRT groups, respectively (Table 3). No treatment-related mortality was recorded.

**Table 2:** Adverse events during chemoradiotherapy

	IC + RT (*n =* 109)	IC + CCRT (*n =* 111)	*P*-value*
Hematologic			
Anemia (grade 3 or 4)	4 (3.7)	12 (10.8)	0.041
Thrombocytopenia (grade 3 or 4)	4 (3.7)	13 (11.7)	0.026
Neutropenia (grade 3 or 4)	7 (6.4)	26 (23.4)	<0.001
Leukopenia (grade 3 or 4)	8 (7.3)	28 (25.2)	<0.001
Non-hematologic			
Stomatitis (mucositis) (grade 3 or 4)	16 (14.7)	33 (29.7)	0.007
Nausea (grade 3 or 4)	2 (1.8)	24 (21.6)	<0.001
Vomiting (grade 3 or 4)	1 (1.0)	22 (19.8)	<0.001
Diarrhea (grade 3 or 4)	1 (1.0)	3 (2.7)	0.627
Dry mouth (grade 3 or 4)	5 (4.6)	8 (7.2)	0.410
Deafness or otitis (grade 1 or 2)	55 (50.5)	73 (65.8)	0.021
Liver dysfunction (grade 1 or 2)	10 (9.2)	26 (23.4)	0.004
Kidney dysfunction (grade 1 or 2)	6 (5.5)	13 (11.7)	0.101
Cycles of concurrent chemotherapy			
One	0	8 (7.2)	
Two	0	103 (92.8)	

*Calculated using the χ² test

**Table 3:** Grade 3-4 of late adverse events

	IC + RT (*n =* 109)	IC + CCRT (*n =* 111)	*P*-value
Symptomatic temporal lobe necrosis	2 (1.8)	2 (1.8)	>0.99^a^
Cranial neuropathy	1 (1.0)	2 (1.8)	>0.99^a^
Eye damage	1 (1.0)	1 (1.0)	>0.99^b^
Ear (deafness/otitis)	4 (3.7)	6 (5.4)	0.769^a^
Bone necrosis	1 (1.0)	1 (1.0)	>0.99^b^
Trismus	1 (1.0)	1 (1.0)	>0.99^b^
Dry mouth	4 (3.7)	5 (4.5)	>0.99^a^
Neck tissue damage	2 (1.8)	3 (2.7)	>0.99^a^

^a^P values were calculated with the Continuity Correction

^b^P values were calculated with the Fisher exact test

## Discussion

In this prematurely terminated phase 3 trial that enrolled only 50% of the planned sample size, we were unable to definitively establish noninferiority of RT alone versus CCRT for PFS. However, the observed data showed numerically comparable 5-year PFS rates (87.0% vs. 80.7%) with a point estimate favoring the RT group, and the safety profile of RT alone was significantly more favorable.

In 2024, a prospective study conducted by Dai et al. [11] from China demonstrated for the first time that RT alone was noninferior to CCRT for patients with LA NPC after IC. The next question to be answered is which subgroup of patients is most likely to benefit from omitting CC. Patients with SD/PD after IC have a poor prognosis, and theoretically, they are clearly not suitable for receiving RT alone. The optimal candidates are naturally those who have achieved CR/PR. Compared with the results reported by Dai et al. [11], both treatment groups in the present study showed a trend toward improved efficacy. Several potential reasons may account for this observation. First, the overall response rate after IC in our study was 100% compared with 82.9–84.2% in their study. Second, the proportion of patients with stage III disease in our study was 64.0% compared with 54.3% in Dai et al.’s study. Third, treatment adherence to induction chemotherapy appeared to be higher in our study, with all patients completing three cycles, whereas the corresponding completion rate in their study was 93.7%.

Several factors may contribute to the noninferiority of RT alone in our study. First, advancements in imaging and radiotherapy technology have played a crucial role. Magnetic resonance imaging (MRI) is gradually replacing computed tomography (CT) for precise staging of nasopharyngeal carcinoma, and IMRT has gradually replaced traditional two-dimensional radiotherapy, becoming a widely used radiotherapy technique [12, 13]. These technologies provide better tumor target conformity, higher target dose coverage, and superior normal tissue protection, thereby reducing the intensity of chemotherapy without compromising efficacy. Second, successful completion of three cycles of IC significantly reduces tumor volume and increases sensitivity to subsequent RT, ultimately decreasing the locoregional recurrence rate [4, 14]. Third, the inclusion criteria for this study required CR/PR after IC. The favorable response to IC indicates good sensitivity to subsequent RT, thus providing a rationale for omitting CC [15].

The present study demonstrated that omission of concurrent chemotherapy significantly improved treatment tolerability. Specifically, removal of concurrent chemotherapy was associated with lower incidences of grade 3–4 hematologic toxicities, including leukopenia/neutropenia, thrombocytopenia, and anemia (all *P* < 0.05). The rates of grade 3–4 non-hematologic adverse events, particularly nausea and vomiting, were also significantly reduced following omission of concurrent chemotherapy (all *P* < 0.05). In addition, eliminating CC can significantly reduce patients’ medical expenses, primarily including the cost of concomitant chemotherapy drugs themselves, as well as the cost of medications and hospitalization required to prevent and treat chemotherapy-related adverse reactions. Lastly, waiving CC can also reduce the incidence of certain radiotherapy-related adverse events, such as stomatitis (*P* < 0.05). The absence of significant differences in late radiation-induced injuries between the two groups is likely attributable to the fact that late toxicities are primarily determined by radiotherapy parameters—specifically, the total radiation dose, fractionation schedule, and irradiated volume—rather than by the addition of concurrent chemotherapy. Because both groups received the same intensity-modulated radiotherapy protocol, comparable rates of late adverse events were observed.

For patients who achieve stable disease (SD) or progressive disease (PD) after induction chemotherapy—a subgroup with a poor prognosis—alternative treatment strategies warrant investigation in future clinical trials. Potential approaches include: (1) switching to non-cross-resistant chemotherapy regimens for subsequent cycles; (2) radiotherapy intensification, such as dose escalation to residual disease or the addition of radiosensitizers; and (3) incorporation of novel agents, including immune checkpoint inhibitors or targeted therapies, in the post-induction setting. Prospective studies specifically designed for this high-risk population are urgently needed.

This study has several limitations. First, the absence of stratification at the time of randomization led to an imbalance in stage distribution and sex composition between the two treatment groups. To explore the potential impact of these imbalances on survival outcomes, we performed a post hoc propensity score matched analysis (Additional file 1: Table S1). After matching, the survival results remained consistent with the primary analysis, showing no significant differences between groups (Additional file 2: Fig. S1). Nevertheless, these imbalances limit the interpretability of the primary findings, and future trials should incorporate stratification by sex and stage at randomization. Although the present study was not designed to evaluate prognostic factors, we observed an imbalance in sex distribution between the two treatment arms (36.7% female in the IC + RT group vs. 20.7% in the IC+CCRT group). Previous studies have demonstrated that gender independently influences survival outcomes in nasopharyngeal carcinoma, with females showing better overall survival and distant metastasis-free survival compared with males [16]. This imbalance may have contributed to the numerically better survival outcomes observed in the IC + RT group, although the difference did not reach statistical significance. Second, all patients included in this study were from endemic areas, which limits the generalizability of the study results. Third, the present study did not incorporate post-induction chemotherapy EBV-DNA status as an enrollment criterion. Accumulating evidence suggests that undetectable EBV-DNA after induction chemotherapy identifies a subgroup with particularly favorable prognosis. Future trials evaluating de-escalation of concurrent chemotherapy should consider mandating undetectable post-IC EBV-DNA as an enrollment criterion to better select candidates most likely to benefit from omission of concurrent chemotherapy. Fourth, quality-of-life outcomes were not compared between the two groups. Although formal quality-of-life (QoL) assessments were not performed in this trial, the observed significant reductions in grade 3–4 nausea, vomiting, and mucositis in the IC + RT group suggest a potential QoL benefit. Lower rates of severe gastrointestinal and oral toxicities are likely to translate into preserved physical functioning, reduced need for supportive care interventions (e.g., parenteral nutrition, opioids, or hospitalization), and improved emotional well-being. Moreover, avoidance of severe acute toxicities may facilitate better long-term recovery and treatment adherence. Future studies should incorporate validated QoL instruments to formally evaluate these potential benefits. Finally, the trial was terminated early due to slow accrual, achieving only 50% of the target sample size. This resulted in insufficient statistical power to draw a definitive noninferiority conclusion. The wide confidence intervals around the PFS difference preclude any robust claim of noninferiority or equivalence.

## Conclusions

In this prematurely terminated phase 3 noninferiority trial that enrolled only 50% of the planned sample size, we were unable to draw a definitive conclusion regarding the noninferiority of IC + RT versus IC+CCRT for PFS in NPC patients achieving CR/PR after IC. Nevertheless, several key findings emerged. First, IC + RT was associated with significantly lower rates of grade 3–4 hematologic and gastrointestinal toxicities, indicating improved tolerability. Second, the observed 5-year PFS rates (87.0% vs. 80.7%) were numerically comparable, with the point estimate falling within the prespecified noninferiority margin of 10%. However, the wide confidence intervals attributable to the limited sample size preclude any robust statistical claim of noninferiority or equivalence. Therefore, these findings should be considered hypothesis-generating rather than confirmatory. These hypothesis-generating findings provide a rationale for future adequately powered trials.

## Supplementary Information

Below is the link to the electronic supplementary material.

Supplementary Material 1: Additional file 1: Table S1.

Supplementary Material 2: Additional file 2: Figure S1.

## Data Availability

The datasets generated and/or analysed during the current study are not publicly available due to the protection of participant privacy and institutional data protection policies. Data are available from the corresponding author upon reasonable request, subject to approval from the institutional ethics committee of Zhejiang Cancer Hospital and execution of a signed data access agreement. Requests should be directed to the corresponding author (Prof. Xiao-Zhong Chen, email: chenxiaozhong2016@163.com), who will respond within 30 days. Restrictions on data reuse, including prohibition of redistribution and requirement for co-authorship acknowledgment where substantial intellectual contribution is made, will be specified in the data use agreement.

## Abbreviations

ALT: Alanine aminotransferase
AST: Aspartate aminotransferase
CC: Concomitant chemotherapy
CCRT: Concurrent chemoradiotherapy
CR: Complete response
CT: Computed tomography
DMFS: Distant metastasis-free survival
EBV: Epstein-Barr virus
IC: Induction chemotherapy
IMRT: Intensity-modulated radiotherapy
LA: Locally advanced
LRRFS: Locoregional relapse-free survival
LRFS: Local relapse-free survival
MRI: Magnetic resonance imaging
NCCN: National Comprehensive Cancer Network
NPC: Nasopharyngeal carcinoma
OS: Overall survival
PD: Progressive disease
PFS: Progression-free survival
PR: Partial response
RRFS: Regional relapse-free survival
RT: Radiotherapy
SD: Stable disease
