# Supplementary material for: Randomized, multicenter phase 3 study evaluating radiotherapy versus concurrent chemoradiotherapy in nasopharyngeal carcinoma patients achieving CR/PR after induction chemotherapy
Source: BMC Med. 2026 Jul 11;24:550. doi: 10.1186/s12916-026-05064-8 (PMC13644246; doi:10.1186/s12916-026-05064-8)
Supplement: Supplementary file 1 [file 12916_2026_5064_MOESM1_ESM.docx]

Table S1. Basic characteristics after propensity score matching. The table presents baseline demographic and clinicopathological characteristics of 77 matched pairs from the IC+RT and IC+CCRT groups, including age, sex, EBV-DNA level, T stage, N stage, clinical stage, and response evaluation after induction chemotherapy. All variables were well balanced between the two groups after matching (all P > 0.05).

| Characteristic | IC+RT  N=77 | IC+CCRT  N=77 | P value |
| --- | --- | --- | --- |
| Age, years | 54.0 (23.0–67.0) | 56.0 (20.0–75.0) | 0.5 |
| Sex |  |  | >0.9 |
| Female | 20 (26.0%) | 20 (26.0%) |  |
| Male | 57 (74.0%) | 57 (74.0%) |  |
| EBV DNA |  |  | 0.4 |
| >500 | 24 (31.2%) | 30 (39.0%) |  |
| 0-500 | 53 (68.8%) | 47 (61.0%) |  |
| T stage |  |  | >0.9 |
| T1-2 | 8 (10.4%) | 7 (9.1%) |  |
| T3-4 | 69 (89.6%) | 70 (90.9%) |  |
| N stage |  |  | >0.9 |
| N0-1 | 39 (50.6%) | 39 (50.6%) |  |
| N2-3 | 38 (49.4%) | 38 (49.4%) |  |
| Clinical stage |  |  | 0.9 |
| III | 50 (64.9%) | 52 (67.5%) |  |
| IV | 27 (35.1%) | 25 (32.5%) |  |
| Response evaluation |  |  | 0.2 |
| CR | 4 (5.2%) | 10 (13.0%) |  |
| PR | 73 (94.8%) | 67 (87.0%) |  |
