# Supplementary material for: Randomized, multicenter phase 3 study evaluating radiotherapy versus concurrent chemoradiotherapy in nasopharyngeal carcinoma patients achieving CR/PR after induction chemotherapy
Source: BMC Med. 2026 Jul 11;24:550. doi: 10.1186/s12916-026-05064-8 (PMC13644246; doi:10.1186/s12916-026-05064-8)
Supplement: Supplementary file 2 [file 12916_2026_5064_MOESM2_ESM.docx]

Figure S1. Kaplan-Meier estimates of survival outcomes in the propensity score-matched cohort. (A) Progression-free survival, (B) Overall survival, (C) Local relapse-free survival, (D) Locoregional relapse-free survival, (E) Regional relapse-free survival, and (F) Distant metastasis-free survival in patients receiving IC + RT versus IC + CCRT. No significant differences were observed between the two treatment groups for any of the survival endpoints (all P > 0.05), consistent with the primary analysis results.
